# Silicon Influences Soil Availability and Accumulation of Mineral Nutrients in Various Plant Species

**DOI:** 10.3390/plants7020041

**Published:** 2018-05-19

**Authors:** Maria Greger, Tommy Landberg, Marek Vaculík

**Affiliations:** 1 Department of Ecology, Environment and Plant Sciences, Stockholm University, SE 106 91 Stockholm, Sweden; tommy.landberg@su.se; 2 Department of Plant Physiology, Faculty of Natural Sciences, Comenius University in Bratislava, Mlynska Dolina B2, SK 842 15 Bratislava, Slovakia; vaculik.marek@gmail.com

**Keywords:** carrot, lettuce, maize, pea, nutrient accumulation, silicon, soil, uptake, wheat

## Abstract

Silicon (Si) effects on mineral nutrient status in plants are not well investigated. It is known that Si has a beneficial effect on plants under stressed conditions. The aim was to make a state of the art investigation of the Si influence: (1) on nutrient availability in four different soil types, namely clayish, sandy, alum shale and submerged soil; and (2) on accumulation of various nutrients in maize, lettuce, pea, carrot and wheat growing in hydroponics. Soil was treated with K_2_SiO_3_ corresponding to 80 and 1000 kg Si ha^−1^ and the nutrient medium with 100, 500, 1000 and 5000 μM Si. In general, Si effects were similar in all analyzed plant species and in all soil types tested. Results showed that, in soil, Si increased the availability of Ca, P, S, Mn, Zn, Cu and Mo and that of Cl and Fe tended to increase. The availability of K and Mg was not much affected by Si. Uptake from solution of S, Mg, Ca, B, Fe, and Mn increased; N, Cu, Zn and K decreased; P decreased/increased; and Cl and Mo was not influenced. Translocation to shoot of Mg, Ca, S, Mn, and Mo increased; Fe, Cu and Zn decreased; and K, P, N, Cl and B was not affected. It was concluded that, if plants had been cultivated in soil, Si-maintained increased availability of nutrients in the soil solution would probably compensate for the decrease in tissue concentration of those nutrient elements. The study shows that Si also influences the nutrient uptake in non-stressed plants.

## 1. Introduction

The lack of agricultural land is a very serious topic since the human population increases every day. Therefore, it will become important to increase the food production and also to use less suitable soils, such as those with high salinity, poor nutrient availability, low water holding capacity and slightly polluted sites. Lower quality soils have impacts on plants, ending up in lower food production. To improve this situation, the use of silicon (Si) has been widely discussed in recent years, since Si makes plants more resistant against salinity [1], decreases uptake of some toxic elements [2,3,4], and increases the biomass of food crops [5,6]. The mineral nutritional value of crops is also important to consider. Whether Si influences the uptake and accumulation of various plant nutrients is, however, less investigated, especially in plants other than rice and sugar canes, which are Si accumulators and for which Si is beneficial.

Silicon is the second most common element in earth’s crust. However, not all Si in soil is available to plants; most of it is locked up in recalcitrant silicate minerals and only a much smaller fraction is available for plants [7]. The soluble fraction of Si is redox and pH dependent [8]. Once absorbed in plants, Si forms solid-phase phytoliths, which are recycled to the soil solution with the decay of dead plant material and may again be absorbed by plants [9].

Silicon is taken up by plants in the form of undissociated silica acid [10] and is translocated in the same form through the xylem [11,12]. The uptake is thought to be passive [13]. In recent years, several Si transporters in root exo- and endodermis have been discovered [14,15,16,17]. Silicon is found in higher levels primarily in monocotyledons, especially grasses (0.3–1.2% of DW), with very high content in rice (up to 10%) [10,18,19]. In plant tissues, Si is often found as hydrogen bound Si–organic complexes [10] and impregnates the walls of epidermis and vessels [20], where its role is in strengthening plant tissues and reducing water transpiration and fungal infections. Silicon associates with cell wall components, including polysaccharides, lignins and proteins [21]. The majority of Si is present in plants in the form of hardly dissolved phytoliths [22].

Silicon affects the uptake, distribution and functionality of several mineral nutrients in plants. According to the literature, among the macronutrient elements, nitrogen (N), phosphorus (P), potassium (K), magnesium (Mg) and calcium (Ca) are influenced in different, not unified, ways [23,24,25,26,27,28,29,30,31,32,33,34,35]. Among micronutrients, boron (B) and manganese (Mn) seem to be most strongly influenced by Si [23,36,37,38,39,40]. Silicon also influences Fe, Cl and Zn uptake [23,24,26,39,41,42,43,44,45,46]. Due to various Si effects, it might be possible that Si modifies uptake and acquisition of nutrients differently between various plants species. 

To understand the heterogeneity of Si effects on nutrient uptake, it is important to distinguish between studies made in soil and those made in hydroponics. In soil, Si might affect the binding of the nutrient elements to soil particles making them more or less available for plant uptake. Silicon is known to reduce the soil sorption of P, especially at low pH, and, thus, increases the plant-available portion of P in the soil [47,48]. Phosphorus is sorbed mainly onto Fe-, Al- and Mn-hydroxides in the soil. Silicon associates with Fe(III) and Mn and, thus, reduces the pool of hydroxides and change the availability of Fe and Mn [2,49].

Until now, a general assembly of Si effects on uptake, distribution and status of various essential nutrients is missing. The aim of the present work was to investigate the effects of Si on uptake and distribution of various mineral nutrients in five various plant species and together with literature review obtain a general overview of Si effects on plant nutrient status. We investigated and compared different plant types: monocotyledons (wheat and maize), eudicotyledons (carrot, pea, lettuce), silicon accumulator (wheat), cereals (wheat, maize), C4 plants (maize), leafy vegetables (lettuce), nitrogen fixating plant (pea), and root vegetable (carrot). The type of cultivation media may influence both soil plant availability and uptake from soil solution as well as distribution within the plants. We therefore compared influence of Si on: (1) nutrient availability in different soil types; and (2) nutrient uptake in plants from nutrient solution.

## 2. Materials and Methods

In this work, the effect of Si on the availability of various mineral elements in four different soil types were investigated. Additionally, influence of Si on accumulation and distribution of nutrients was analyzed in five different plant species, cultivated in nutrient medium, and performed at different time length.

### 2.1. Soils

About 10 L each of four soils having different properties (Table 1) were collected from field:

(1) Clay soil was collected from a farmland at Brunnby farm in Västerås, Sweden (N 59°36.99′, E 16°39.29′).
(2) Sandy soil was collected from a farmland at Torslunda field station at Öland, Sweden (N 56°37.86′, E 16°30.62′).
(3) Alum shale soil was collected from a farmland at Kinne-Kleva, Västra Götaland, Sweden (N 58°34.33′, E 013°26.19′).
(4) Submerged soil was collected close to a small river in Adak mine site, Lappland, Sweden (N 65°20.94′, E 18°35.59′).

Soil samples, each of 150 g, were placed in 120 mL plastic pots with lid and Si in the form of K_2_SiO_3_ was thoroughly mixed into the soil in three various concentrations corresponding to 0, 80 and 1000 kg Si ha^−1^. Samples were treated for 90 days at room temperature and soil was kept at field capacity, which were checked once a week. The experiment was performed in five replicates.

In an additional experiment with similar set up, alum shale, clay and sandy soil was treated for six months with 0, 80, 160 and 500 kg Si ha^−1^ using amorphous SiO_2_ (Microsilica from Elkem). This was done to eliminate the K addition along with the addition with K_2_SiO_3._

At the end of the treatment, several soil parameters were analyzed. pH was analyzed after extraction of soil in distilled water (1:1; *w*:*w*) for 2 h [50]. Conductivity was analyzed after extraction of soil in distilled water (1:5; *w*:*w*) for 1 h [50]. Cation exchange capacity (CEC) was analyzed according to ISO 11260:1994 [51]. Organic matter content was measured as weight of loss-in-ignition at 550 °C for 2 h [50]. Clay and sand was determined according to Bowman and Hutka [52]. 

### 2.2. Plant Materials and Cultivation

In this work, five various plant species were compared:

maize (*Zea mays* L. cv. Reduta);
lettuce (*Lactuca sativa* L. cv. Amerikanischer brauner);
spring bread wheat (*Triticum aestivum* cv. Tjalve);
carrot *(Daucus carota* cv. Nantaise); and
pea (Pisum sativum cv. Fenom).

Prior to germination, seeds were soaked in distilled water for 4 h. Germination was performed in darkness and room temperature. Seeds were germinated in vermiculite, except maize, which was germinated in rolled filter paper. The time of germination, when the radicle was seen, was different for the species: 3 days for maize, 7 days for lettuce, 5–7 days for wheat, and 8 days for pea. After germination, the most viable seedlings were transferred to styrophoam plates, which was placed in pots (1–2.3 L) containing nutrient medium. In each pot, 4 (wheat, maize, pea, and lettuce) or 10 (carrot) plants were cultivated. The number of pots (i.e., replicates) was in the case of three-week treatment 4 (wheat, lettuce, carrot, and pea) and in the short-term treatment 5 (maize and lettuce) or 10 (wheat). The nutrient media had different composition for different plant species (Table 2). All nutrient media were prepared as half-strength (50%) and pH was adjusted to 6.2–6.5.

### 2.3. Silicon Treatment

The Si treatments were always in the form of K_2_SiO_3_ with the following concentrations and time:

Experiment 1 (E1): Maize was treated with 0 or 5000 μM Si during 7 days.
Experiment 2 (E2): Wheat and lettuce were treated with 0, 100, 500 or 1000 μM Si for 5 days.
Experiment 3 (E3): Wheat, lettuce, carrot and pea were treated with 0, 100, 500 or 1000 μM Si for 3 weeks.

Maize plants in E1 were cultivated in a climate chamber equipped with HQI-BT 400W OSRAM lamps giving 300 μmol m^−2^ s^−1^ PAR and 16 h photoperiod at 25/23 °C, day and night temperature regime and the humidity of 80%. Nutrient medium was changed every second day. After seven-day treatment, plants were harvested, divided into shoots and roots and dried at 70 °C for 72 h.

Lettuce and wheat plants in E2 were grown in a climate chamber equipped with HQI-BT 400 W OSRAM lamps giving 270 μmol m^−2^ s^−1^ PAR with 16 h photoperiod at 23 °C/19 °C day/night temperature regime and the humidity of 80%. Water loss was compensated daily. After five-day treatment, plants were harvested, divided into shoots and roots and dried at 80 °C for 48 h.

Carrot, pea, lettuce and wheat plants in E3 were grown in a climate chamber equipped with HQI-BT 400W OSRAM lamps giving 270 μmol m^−2^ s^−1^ PAR with 16 h photoperiod at 23 °C/19 °C day/night temperature regime and the humidity of 70%. Water loss was compensated daily. Nutrient medium was changed once a week. After three-week treatment, plants were harvested, divided into shoots and roots and dried at 105 °C for 24 h.

### 2.4. Analysis of Elements in Plants and Soils

Dried plants were milled and then the samples were prepared differently for determination of various elements. For Cl and S analysis the plant material was ashed at 500 °C in a muffle furnace (Furnace Type 6000, Tourmaline), and for Cl the ash was dissolved in conc. HNO_3_ and for S the ash was boiled in carbonate/bicarbonate buffer (2.6 mM and 2.4 mM of Na_2_CO_3_ and NaHCO_3_, respectively) for 3 h. For the determination of Si, K, Mg, Ca, Mn, Fe, Cu, Mo, B, and Zn, the plant material was wet digested with HNO_3_:HClO_4_ (7:3).

Soils leachates were prepared with different extraction methods depending on the analyzed elements. All methods are considered to give concentrations, which resemble the available fraction of particular element in the soil. Thus, for K, Mg, Ca, Mn, Fe, Cu, Mo, Cl and Zn determination, the soil samples were extracted with 1 M NH_4_Ac for 16 h (1.0 g of soil per 50 mL for 20 h) at one pH-unit lower than the soil pH [56]. For P determination, the soil samples were extracted using 0.02 M lactate (1.0 g of soil in 20 mL for 120 min) [57]. For Si and S determination, the soil samples were extracted with 0.01 M CaCl_2_ (1.0 g of soil in 50 mL for 30 min) [58]. 

The analyses of the elements were performed in the same way for plants and soils. Phosphorus was spectrophotometrically analyzed according to Swedish Standard (SIS 028126) using ammonium molybdate and potassium antimony tartrate. Nitrogen (plants only) was analyzed with elemental analyzer (CHN-900, LECO Corp., St. Joseph County, MI, USA). Chlorine and S were analyzed according to the procedures of Egner et al. [57], Edeogu [59] and Tack et al. [60] (based on AOAC 1984 [61]); Cl was precipitated using 0.1 M AgNO_3_, Ag was then analyzed by atomic absorption spectrophotometer (AAS; SpectrAA 55b, Varian Inc., Palo Alto, CA, USA), S was precipitated using BaCl_2_ and Ba was analyzed by AAS. Silicon, K, Mg, Ca, Mn, Fe, Cu, Mo, B, Zn were analyzed by AAS. Certified reference plant material (GBW07604) from Institute of Geophysical and Geochemical Exploration, Langfang, China, was used in all analyses.

### 2.5. Calculations and Statistical Analysis

The root and shoot concentrations of various elements are given. From those the net accumulation via roots was calculated as:

(1) $$\mathrm{Net}\;\mathrm{accumulation}\;\mathrm{of}\;X\;\left(\mu g\;g\;\mathrm{DWroot}\right)=\frac{\mathrm{Total}\;\mathrm{amount}\;\mathrm{of}\;X\;\mathrm{in}\;\mathrm{the}\;\mathrm{whole}\;\mathrm{plant}}{\mathrm{Root}\;\mathrm{weight}}$$

where X is the element in question.

The investigation was performed with 4–10 pots, with different number (4–10) of plants in each pot. One replicate consisted of materials from all plants in each pot. Statistical analysis of the data was performed using Student’s *t*-test and one-way ANOVA with Statistica 4.1 (Statsoft Inc, St Tulsa, OK, USA). JMP 10 (SAS Institute Inc., Cary, NC, USA) was used to identify differences between controls and Si treatments. Spearman correlation tests were performed in some tests to avoid non-linear differences. For PCA-analysis the package R was used. Significance level was set to *p* = 0.05.

## 3. Results

### 3.1. Soil

Silicon added in concentration of 1000 kg Si ha^−1^ significantly increased the pH with 0.29–0.47 pH-units in all soils while the lower Si addition had no effect on pH (Table 1). None of the other soil parameters analyzed changed with addition of Si.

**Figure 1:**
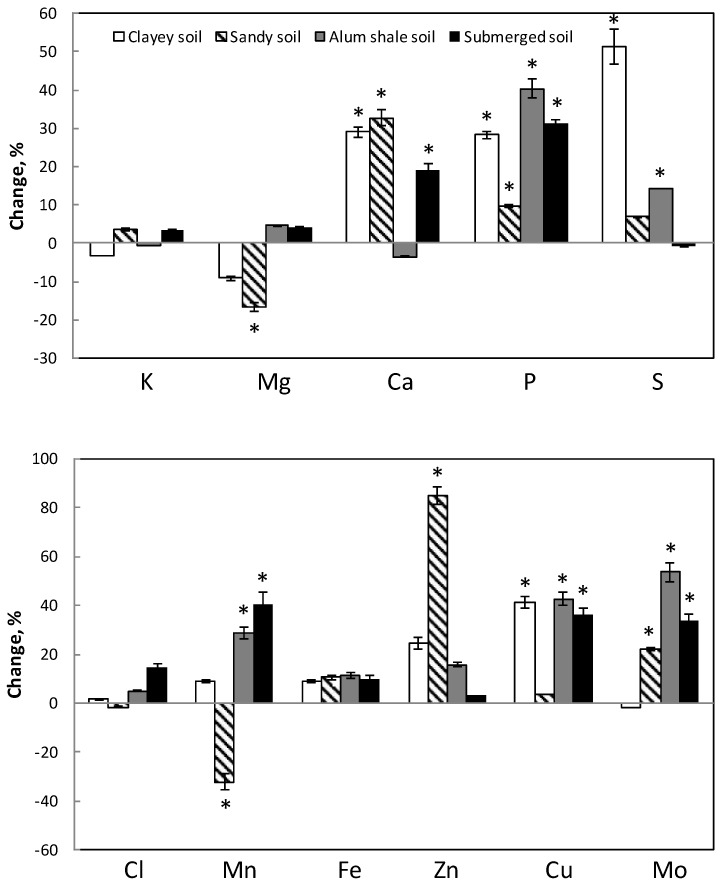
Changes in available concentration of various macro- and micronutrients in Si-treated soils compared with non Si-treated soils. The four different soils were treated with K_2_SiO_3_ corresponding to 1000 kg Si ha^−1^ for 90 days. *n* = 5, ± SE. * indicates significant difference between treated and non-treated soil.

Silicon affected the available concentration of all analyzed elements (i.e., extractable fraction) in the soils to various extents (Figure 1). In general, the availability of Ca, P, S, Mn, Zn, Cu and Mo increased, and that of Cl and Fe tended to increase. Potassium (K) and Mg were not much and not unidirectional affected. The same was shown for K when Si was added as amorphous SiO_2_ (Microsilica; Table 3). Treatment with 1000 kg Si ha^−1^ increased the availability of P 10–40%, S 0–51%, Ca 0–33%, Cu 10–40%, Mo 0–54%, Mn 9–41%, Zn 3–90%, Fe ≈ 10%, and Cl 0–15% (Figure 1). In most cases, addition of 80 kg Si ha^−1^ gave the same effect but lower and not significant. However, the results often show a significant correlation between Si-treatment strength and elements availability.

Comparing soil types, there was no unambiguous difference in the influence of Si on availability of elements; only availability of Mn and Mg decreased significantly in sandy soil (Figure 1).

### 3.2. Plants

Shorter Si treatment (E1 and E2) insignificantly increased maize biomass, although root and shoot biomass was not affected or even decreased in wheat and lettuce, respectively (not shown). Longer Si treatment (E3) increased shoot biomass of all investigated plant species (non-significant increase only for pea), while oppositely, root biomass was not affected and significantly increased only in pea (not shown).

The concentration of Si was four times higher in maize than in lettuce and wheat in (E1 and E2) untreated plants (Table 4). When treated with Si the Si concentration increased more in roots than in shoots. With 5000 μM Si the concentration of this element in maize shoot and roots increased 85 and 128 times, respectively, while, at 1000 μM Si, the Si concentration increased in lettuce 34 and 57 times and in wheat 147 and 300 times in shoot and roots, respectively. In addition, when plants were treated with Si for three weeks (E3), the Si concentration increased more in roots than in shoots. With 1000 μM Si treatment the Si concentration increased in wheat 200 and 234 times and in lettuce 34 and 60 times in shoots and roots, respectively. In carrot, the Si concentration increased 27 and 50 times, and, in pea, 33 and 70 times in shoots and roots, respectively.

**Figure 2:**
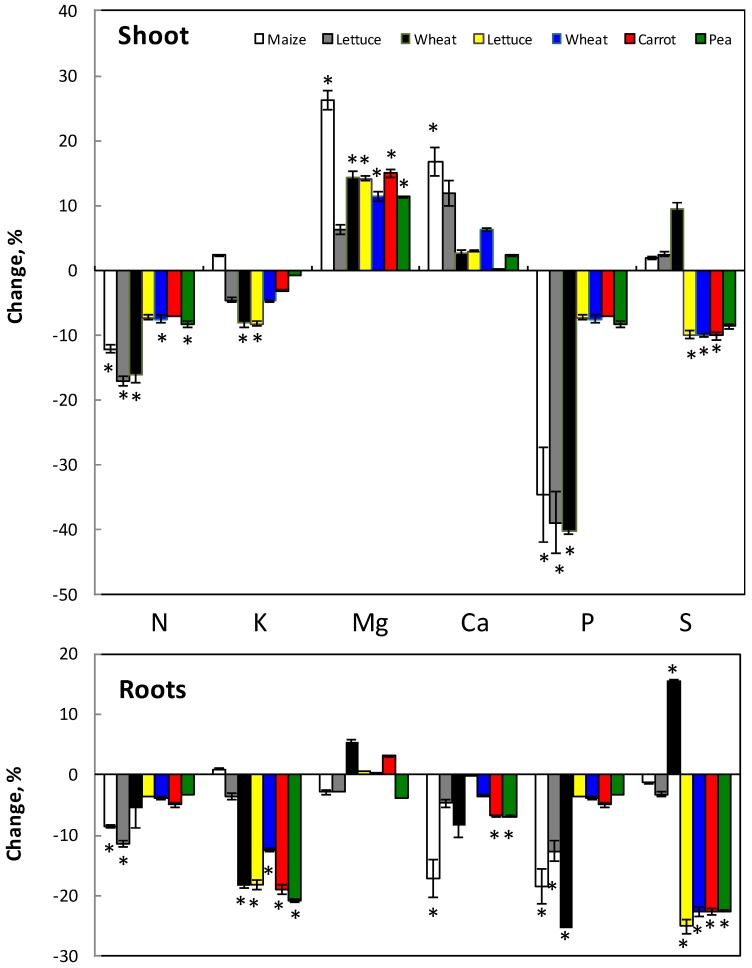
Changes in the concentration of various macronutrients in roots and shoots of Si-treated plants compared with concentrations in non-treated plants. Bars in black and white shows maize, lettuce and wheat treated with K_2_SiO_3_ corresponding to 1 mM Si for five days (wheat and lettuce) and 5 mM Si for seven days (maize). *n* = 5 (maize and lettuce) and 10 (wheat) ± SE. Bars in color show lettuce, pea, carrot and wheat treated with K_2_SiO_3_ corresponding to 1 mM Si for three weeks. *n* = 4, ± SE. * indicates significant difference from the control.

The concentration of macronutrients in shoots and roots was analyzed and generally the pattern was as follows (Figure 2). Silicon decreased the concentration of N, K, Ca, P and S in roots while that of Mg was not significantly changed among the species. In shoots, however, N, K, P and S decreased while that of Mg and Ca increased in the presence of Si. There was an effect with the duration of the treatment where S decreased with time while P and N decrease is less pronounced at longer treatment in both roots and shoots. The same effect has been shown when using Na_2_SiO_3_ (not shown).

**Figure 3:**
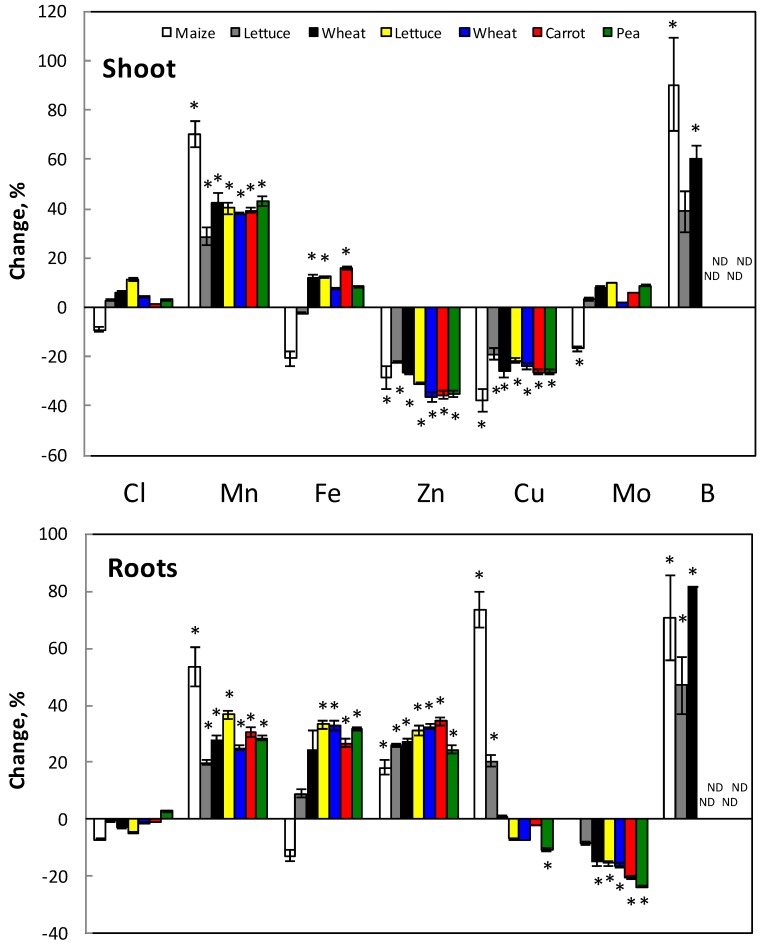
Changes in the concentration of various micronutrients in roots and shoots of Si-treated plants compared with concentrations in non-treated plants. Bars in white, grey and black show lettuce, wheat and maize treated with K_2_SiO_3_ corresponding to 1 mM Si for five days (wheat and lettuce) and 5 mM Si for seven days (maize). *n* = 5 (maize and lettuce) and 10 (wheat) ± SE. Bars in color show wheat, carrot, lettuce and pea treated with K_2_SiO_3_ corresponding to 1 mM Si for three weeks. *n* = 4, ± SE. * indicates significant difference from the control.

The concentration of micronutrients in shoots and roots was analyzed and generally the pattern was as follows (Figure 3). In roots, Mn, Fe, Zn and B increased with Si treatment while Mo decreased and Cl was not affected. In short time treatment, Cu increased while at long time treatment Cu decreased in roots. In shoot, Mn, Fe and B increased with Si treatment while Cu and Zn decreased. The same effect has been shown when using Na_2_SiO_3_ (not shown).

**Figure 4:**
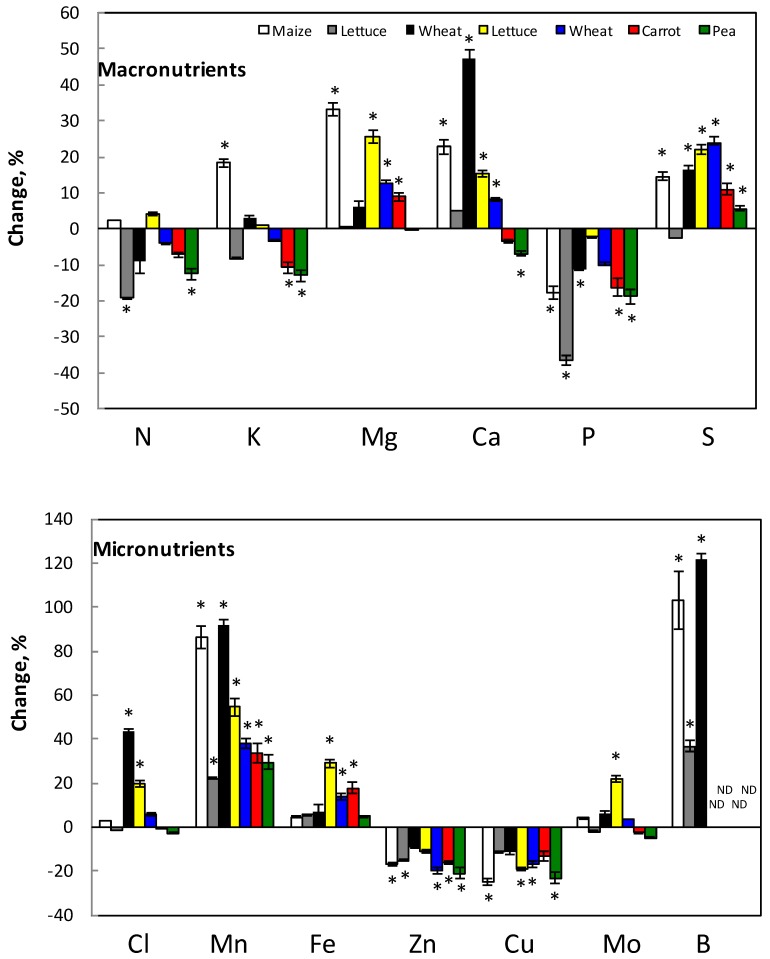
Changes in the net accumulation of various macro- and micronutrients in roots and shoots of Si-treated plants compared with concentrations in non-treated plants. Bars in black and white shows maize, lettuce and wheat treated with K_2_SiO_3_ corresponding to 1 mM Si for five days (wheat and lettuce) and 5 mM Si for seven days (maize). *n* = 5 (maize and lettuce) and 10 (wheat) ± SE. Bars in color show lettuce, pea, carrot and wheat treated with K_2_SiO_3_ corresponding to 1 mM Si for three weeks. *n* = 4, ± SE. * indicates significant difference from the control.

Net accumulation of Si, i.e., accumulation into the whole plant in relation to the root biomass, increased most in wheat, with up to 209 times and 37, 43 and 44 times in carrot, lettuce and pea, respectively (Table 1). Silicon treatment increased the net accumulation for Mg, Ca and S; decreased for P; tended to decrease it for N; and had no general effect on K (Figure 4). In the case of micronutrients, the net accumulation increased for Mn, Fe and B, decreased for Zn and Cu and showed no general trend in the case of Cl and Mo. The same effect has been shown when using Na_2_SiO_3_ (not shown).

## 4. Discussion

The present work was performed to find out if and how Si influences the uptake and distribution of the various nutrient elements in plants. The effect of Si was both by influencing the available fraction of elements in various soil types (Figure 1) as well as direct effect on the nutrient uptake from nutrient solution (Figure 4), which mimicked a soil solution. Within the plant, the distribution of nutrients between root and shoot was also affected (Figure 2 and Figure 3).

Each of the elements seems to have its own relation to Si, and, therefore, no general conclusion can be drawn for all nutrients. Similar conclusion can also be drawn from the work by Islam & Saha [23]. In general, in all investigated plant species in our study, similar effects of Si on nutrient accumulation were found. The treatment period (5–7 days and three-week treatment) did not have any important effect on the nutrient uptake and distribution. However, there are a few exceptions: S decreased with time while P and N decrease were less pronounced at long-term than short-time treatment in both roots and shoots. The applied Si concentration also played a role; the higher concentration of Si induced the more pronounced effect. Mostly, only the highest applied concentration of Si, i.e., 1000 kg ha^−1^ and 1000 μM Si lead to significant effects.

Silicon increased the dry biomass at the highest applied concentration and most pronounced in the long-term experiment, although fresh biomass was not influenced (not shown). A likely reason is that the carbohydrate production was enhanced [62]. Silicon is shown to increase the phosphorylation of sugars which in turn promote carbohydrate synthesis [5,6,32,63].

### 4.1. Macronutrients

The net accumulation of N in whole plants as well as root and shoot N concentration slightly decreased in most cases (Figure 2 and Figure 4), however, less pronounced after the long-term treatment. The decrease in N concentration might be due to the increase in growth (not shown) giving a dilution effect. Islam and Saha [23,34] also found reduced N uptake in rice by Si except of very low Si levels (about 0.3 mM), which oppositely increased the N uptake.

The net accumulation of P decreased with addition of Si (Figure 4) which decreased P concentration in both roots and shoots (Figure 2). The present study is not corroborating to other studies showing that P uptake increased by Si [23,32,35]. However, P uptake will most likely increase at plant cultivation in soil by Si addition since Si increased the P availability up to 50% in soils (Figure 1) while the decrease in uptake of P from hydroponic solution is less than 10% (Figure 4). Increase of P uptake by Si in soil has been observed in rice and maize [48,64].

Sulfur net accumulation increased with Si (Figure 4), although the tissue concentration of S decreased with Si in both roots and shoots about 20 and 10%, respectively. This might be explained by Si-enhanced shoot growth and corresponding dilution effect of S concentration in the tissues. The content of S in the shoot may therefore increase and be a reason for the increased net accumulation (Figure 4). Miyake [26] and Thangavelu and Chiranjivi Rao [41] found that S content in plants increased by addition of Si, especially under salt stress. Studies have shown the beneficial role of Si on plants under mineral stress [31,65,66]. Our results showed that S concentration in the tissue decreased with time of treatment (Figure 2). However, since the available S in soil increased in some cases up to 50% after Si addition (Figure 1), it is possible that plant S concentration would be higher when grown in Si fertilized soils.

Potassium net accumulation varied between plant species; however, generally it decreased with Si in most cases (Figure 4). Potassium concentration decreased more in roots than in shoot (Figure 2). This might be due to less effect on the translocation than uptake of K. Since K was added extra along with Si source, the Si effect on the K uptake might be even higher if the extra K (in K_2_SiO_3_) was not added. In rice, Islam and Saha [23] showed that Si reduced the K concentration while according to Miyake [26] it seems that species with high Si accumulation capacity in addition have high uptake of K. Interestingly, rice is a high Si accumulating species. In our case, wheat, which is also Si accumulator, did not have decreased K in the roots. The decrease of K in plant tissues will not be compensated by higher K availability with Si if plants were grown in soil, since Si did not change the K availability much in any of the investigated soils (Table 3 and Figure 1).

The Mg net accumulation increased with Si addition (Figure 4) and this increase was found as an enhanced Mg concentration in the shoot while no change was found in the roots (Figure 2). According to the literature, Mg content in plants increases by Si; however, at salt stress, Mg content decreases by Si [23,27,28,30]. Only in sandy soil, Mg availability decreased significantly at the highest Si addition; otherwise the Mg availability was not significantly affected by Si (Figure 1). Thus, the Mg concentration pattern in roots and shoot, caused by Si, would probably not change if plants were cultivated in the soil.

Calcium uptake increased with Si from solution, especially after short-term Si treatment (Figure 4), and most likely also from soil, since the availability of Ca increased with Si, except from alum shale soil (Figure 1). The Ca distribution in the plant, show that Ca decreased in roots while increased in shoot (Figure 2). Our results did not always corroborate with results from the literature, showing that the Si influence might depend on plant species and cultivation procedure. In cucumber, Ca slightly increased in roots while increased in upper leaves and decreased in lower leaves when treated with Si [65]. No influence of Si on Ca was found in barley shoot under salt stress [67]. Calcium decreased in leaves of common reed [29] with increasing Si addition.

### 4.2. Micronutrients

In this study, there was no general influence of Si on Cl concentration or Cl net accumulation in the plants (Figure 3 and Figure 4). Soil cultivation will most likely not change the Si effect on the Cl concentration in roots and shoot since Cl availability in soil was not changed either (Figure 1). Other works show that Cl uptake decreases by Si, especially at salt stress [26,41].

Boron is the element that was most strongly affected by Si in our study. Increased B net accumulation (Figure 4) and B concentration was observed in all plant parts (Figure 3) but Si had no influence on the distribution of B between roots and shoots (Figure 3). Boron is strongly affected by Si as both these elements share chemical similarities and pathways [37,39]. There are reports indicating both reduced and increased uptake of B by Si [68]. Boron uptake seems to be stimulated by Si in Si accumulators [39]; and wheat, which is a Si accumulator, also had the highest B net accumulation change in the presence of Si of the tested plant species in our study (Figure 4).

The net accumulation of Fe in plants slightly increased in the presence of Si (Figure 4) mirrored as increased Fe concentration in both roots (20–40%) and shoots (10%) (Figure 3). This might be due to that Si prevents translocation of Fe to shoot and thereby more Fe stays in the roots. According to Islam and Saha [23] and Wallace [24], Si decreased Fe concentration in plants, although Pavlovic et al. [43,44,45] showed that Si alleviates Fe deficiency in cucumber by promoting mobilization of Fe. In the case that plants are cultivated in the soil, the effect is probably the same on the Fe concentration in the tissues, since Si only slightly increased the available Fe in the tested soils (Figure 1).

Silicon increased Mn net accumulation and concentration in plants, more in shoot than in roots (Figure 3 and Figure 4). This means that Si promoted Mn translocation to the shoot more than the uptake of Mn by the plant roots. This corroborates with earlier findings [23,36,38,44]. Except of sandy soil, in which Mn availability decreased in the presence of Si, Si increased the Mn availability in other soil types (Figure 1). Therefore, Mn concentration in plants would increase probably even more if they were grown in soil.

The net accumulation of both Cu and Zn decreased about 20% by Si treatment (Figure 4). The likely reason is the decreased Zn and Cu concentration in shoot up to 40%. Additionally, in the case of three-week treatment, the Cu concentration in roots also decreased (Figure 3). The opposite was found in the case of Zn and in short term treatment for Cu where these elements increased in the roots (Figure 3). This increase, however, is lower than the decrease in shoot (Figure 3). In other investigations one found that the concentration in shoot is reduced while Zn concentration in roots is unchanged [23,24,39,46]. The root-shoot distribution is affected and Si decreased the translocation of Zn to the shoot according to Treder and Cieslinski [2]. It is possible that the decreased Zn translocation to the shoot in our experiments depends on the binding of Zn to Si in the roots, since Si itself showed the same pattern of decreased Si distribution to the shoot after Si addition. No data are available in the literature about the Si effect on Cu content. In the soil, the availability of both Cu and Zn increased at Si supply (Figure 1). A higher uptake, due to higher availability, might, however, not change the Si effect on the distribution of Cu and Zn between root and shoot.

In most cases, Si did not influence Mo net accumulation (Figure 4). Thus, Si rather influenced the distribution in the plant and not the uptake since Mo concentration in roots decreased, especially at high Si addition, while in shoot it slightly increased (Figure 3). Except for clayey soil, it is possible that cultivation in soil would increase Mo concentration in both roots and shoots, since Si increased the available Mo concentration in soil up to 50% (Figure 1). There are, to our knowledge, no data about the influence of Si on Mo concentration in plants and soil in the literature.

Possible reasons why Si increases the concentration of some elements could be that Si promotes binding of them in plant tissues. If this occurs in the roots but not in the shoots, Si affects the translocation of elements to the shoot. This could be the case of Zn and Cu. It is also known that Si can influence the development of apoplasmic barriers in roots, controlling the apoplasmic pathways and followed translocation via root apoplasm to the shoot [3,68,69,70,71].

## 5. Conclusions

Silicon influenced the uptake and accumulation of several macro- and micronutrients in various plants and plant organs. However, differences in the uptake and distribution of individual elements and plants indicated that Si affected the macro- and micronutrients within plant tissues individually. Therefore, no general conclusion about the effect of Si on nutrients uptake can be postulated. These differences might be related with the concentration of Si in the medium or with restricted translocation of these elements via binding to silicates within various plant tissues or regulating the apoplasmic transport pathways. Even though Si showed decreased concentration of various nutrients in plant tissues, this decrease could be minimized by cultivation in soil where the availability of the elements increases by Si addition.

There is also a suggestion that Si addition enhance translocation of transport molecules, such as citrate, which may contribute to the metal transport from root to shoot, and will diminish deficiency symptoms [40]. Another suggestion is that Si only helps plants during stress situations, such as increase nutrient uptake during nutrient deficiency [31,65]. In the present investigation, plants did not have any nutrient deficiency and were in a non-stressed state. This means that Si may influence the nutrient uptake also in non-stressed plants.

## Figures and Tables

**Table 1:** Properties of the investigated soils before and after 90 days treatment with various supplementations of Si. *n* = 5, ± SE. * indicates significant difference *p* < 0.05 from the untreated soil.

Soil Type	Si-Treatment	pH	Conductivity	CEC	Org. Content	Clay	Sand
	kg ha^−1^		ms	mmol/gDW	%	%	%
Clayey	0	6.52 ± 0.04	12.7 ± 0.4	0.12 ± 0.01	6.07 ± 0.29	17.9 ± 1.2	41.3 ± 2.4
	80	6.52 ± 0.01	12.1 ± 0.6	0.12 ± 0.00	5.62 ± 0.33	19.4 ± 1.6	41.0 ± 2.0
	1000	6.95 ± 0.05*	11.9 ± 0.7	0.13 ± 0.01	5.91 ± 0.23	17.3 ± 1.5	40.9 ± 1.7
Sandy	0	7.12 ± 0.02	6.4 ± 0.6	0.08 ± 0.00	4.78 ± 0.43	0.2 ± 0.0	65.4 ± 4.1
	80	7.20 ± 0.02	6.0 ± 0.2	0.08 ± 0.01	5.30 ± 0.36	0.2 ± 0.0	66.3 ± 4.7
	1000	7.41 ± 0.01*	6.0 ± 0.4	0.09 ± 0.01	4.88 ± 0.13	0.2 ± 0.0	66.0 ± 4.0
Alum shale	0	7.64 ± 0.03	18.8 ± 1.4	0.21 ± 0.00	10.28 ± 0.42	4.1 ± 0.2	50.4 ± 2.2
	80	7.62 ± 0.07	16.9 ± 1.3	0.21 ± 0.48	9.64 ± 0.81	3.9 ± 0.1	51.4 ± 2.8
	1000	7.93 ± 0.06 *	18.1 ± 0.7	0.21 ± 0.02	10.36 ± 0.03	4.1 ± 0.1	51.4 ± 3.1
Submerged	0	6.84 ± 0.04	17.5 ± 1.5	0.17 ± 0.00	6.53 ± 0.49	3.2 ± 0.3	55.1 ± 4.0
	80	6.80 ± 0.05	16.8 ± 0.1	0.16 ± 0.00	5.81 ± 0.44	3.3 ± 0.2	54.9 ± 4.6
	1000	7.31 ± 0.04 *	18.0 ± 1.3	0.17 ± 0.79	5.81 ± 0.31	2.8 ± 0.1	55.6 ± 4.6

**Table 2:** Concentration of nutrients in full strength (100%) nutrient media used in the present study. Columns 2–4 are for maize, lettuce and wheat treated for 4–5 days with silicon. All plant species cultivated for three weeks with Si were grown in Hoagland nutrient medium (Column 5). For comparison, literature data on concentration in soil solution of the various nutrient elements are indicated.

Nutrient	Maize	Lettuce [53]	Wheat	Hoagland Medium [54]	Soil Solution [55]
	μM	μM	μM	μM	μM
NO_3_	7500	16,000	16000	14,000	750
NH_4_	0.16	1000	1000	1000	100
K	2700	11,500	11,500	6000	250
Ca	2500	3000	3000	4000	250
Mg	1000	2000	2000	2000	100
P	200	2000	2000	1000	0.04–25
S	1000	2000	2000	2000	100
Fe	122	25	50	25	2
B	23	10	20	46	3
Mn	4.6	16	16	9	1
Zn	0.38	0.35	0.35	0.8	0.5
Cu	0.16	0.16	0.16	0.3	0.1
Mo	0.25	0.21	0.21	0.1	0.02
Cl	9.2	107	182	18	100

**Table 3:** Changes in available K concentration in Si-treated soil compared with non Si-treated soil. The three different soils were treated with amorphous SiO_2_ (Microsilica) corresponding to 0, 80, 160 and 500 kg Si ha^−1^ for six months. *n* = 4, ± SE. *^X^* significant correlation at *p* < 0.05 between available K and Si treatment. * indicates significant difference between treated and non-treated soil.

Soil Type	Si, kg ha^−1^	% of Control
**Sandy**	0	100
	80	103.0 ± 0.62 *
	160	104.2 ± 0.33 *
	500	104.2 ± 0.48 *
	*r*	0.65 *^X^*
**Clayey**	0	100
	80	99.8 ± 0.42 *
	160	99.5 ± 0.23 *
	500	98.3 ± 0.13 *
	*r*	–0.85 *^X^*
**Alum shale**	0	100
	80	99.6 ± 0.01 *
	160	99.2 ± 0.09 *
	500	98.7 ± 0.08 *
	*r*	–0.83 *^X^*

**Table 4:** Si concentrations in shoots and roots and net accumulation (total uptake of element per g of root DW) of various plant species untreated and treated with K_2_SiO_3_ during 5–7 days or three weeks. *n* = 4 (three-week-treatment), *n* = 5 (maize and lettuce) and 10 (wheat) ± SE. * indicates significant difference from the control. *p*-value indicates if concentration of element increase/decrease with increased Si-treatment.

Species		5–7 Days Treatment			3-Week Treatment		
Si (μM)		Shoot	Root	Net Accumulation	Shoot	Root	Net Accumulation
		μg gDW^−1^		(mg gDW_root_^−1^)	μg gDW^−1^		(mg gDW_root_^−1^)
***Maize***							
	0	120 ± 18	115 ± 17	—	—	—	—
	5000	10294 ± 1544 *	14702 ± 2205 *	30.23 ± 3.15 *	—	—	—
***Lettuce***							
	0	26 ± 6	31 ± 4	—	26 ± 1	31 ± 0	—
	100	—	—	—	96 ± 7 *	762 ± 9 *	1.07 ± 0.09 *
	500	—	—	—	512 ± 16 *	1528 ± 57 *	3.88 ± 0.35 *
	1000	882 ± 132 *	1776 ± 266 *	5.78 ± 0.75 *	887 ± 60 *	1844 ± 165 *	6.21 ± 0.38 *
	*p*	—	—	—	<0.001 *	<0.001 *	<0.001 *
***Wheat***							
	0	24 ± 1	26 ± 5	—	21 ± 2	36 ± 4	—
	100	—	—	—	2486 ± 244 *	3870 ± 152 *	14.05 ± 0.96 *
	500	—	—	—	3711 ± 160 *	7045 ± 451 *	22.66 ± 1.34 *
	1000	3535 ± 157 *	7798 ± 528 *	23.30 ± 1.41 *	4008 ± 44 *	8439 ± 721 *	25.21 ± 1.44 *
	*p*	—	—	—	<0.001 *	<0.001 *	<0.001 *
***Carrot***							
	0	—	—	—	22 ± 2	27 ± 1	—
	100	—	—	—	158 ± 4 *	415 ± 35 *	0.61 ± 0.07 *
	500	—	—	—	344 ± 17 *	977 ± 5 *	1.42 ± 0.14 *
	1000	—	—	—	586 ± 9 *	1364 ± 46 *	2.13+0.24 *
	*p*	—	—	—	<0.001 *	<0.001 *	<0.001 *
***Pea***							
	0	—	—	—	19 ± 2	21 ± 1	—
	100	—	—	—	108 ± 3 *	549 ± 48 *	0.72 ± 0.06 *
	500	—	—	—	355 ± 18 *	1070 ± 71 *	1.73 ± 0.16 *
	1000	—	—	—	641 ± 16 *	1477 ± 48 *	2.69 ± 0.22 *
	*p*	—	—	—	<0.001 *	<0.001 *	<0.001 *

## Supplementary Materials

The following are available online at http://www.mdpi.com/2223-7747/7/2/41/s1, Table S1: Available macronutrient concentration in investigated soils after 90 days treatment with various supplementations of Si., Table S2: Available micronutrient concentration in investigated soils after 90 days treatment with various supplementations of Si. Table S3 Biomass of plants grown in nutrient medium during 5–7 days treatment with or without K_2_SiO_3_. Table S4 Biomass of plants grown in nutrient medium during three weeks treatment with or without K_2_SiO_3_. Table S5 Concentrations of nutrients in shoots and roots of various plant species untreated and treated with K_2_SiO_3_ during 5–7 days. Table S6 Concentration of various nutrients in shoots and roots of various plant species untreated and treated with silicon for three weeks. Table S7 Net accumulation (total uptake of element per g of root DW) of various elements in maize, wheat and lettuce untreated and treated with silicon for 5–7 days. Table S8 Net accumulation of various elements in wheat, carrot, pea and lettuce untreated and treated with K_2_SiO_3_ for three weeks.
