# Supplementary material for: Silicon Influences Soil Availability and Accumulation of Mineral Nutrients in Various Plant Species
Source: Plants (Basel). 2018 May 19;7(2):41. doi: 10.3390/plants7020041 (PMC6027514; doi:10.3390/plants7020041)
Supplement: Supplementary file 1 [file plants-07-00041-s001.pdf]

Supplement — Greger and Landberg

Table S1. Available macronutrient concentration in investigated soils after 90 days treatment with various supplementations of Si. *P*-value indicates if concentration of element increase/decrease with increased Si-treatment. n=5, ±SE. *indicates significant difference $P<0.05$ from the untreated soil.

Soil type	Treatment Si kg ha^{-1}	K mg kg^{-1}	Mg mg kg^{-1}	Ca mg kg^{-1}	P mg kg^{-1}	S mg kg^{-1}
Clayey						
	0	177 ± 8	144 ± 9	1459 ± 59	18.1 ± 0.1	12.7 ± 0.8
	80	184 ± 7	139 ± 9	1475 ± 13	19.5 ± 0.9	13.1 ± 0.7
	1000	171 ± 4	131 ± 4	1883 ± 54*	23.2 ± 0.8*	19.2 ± 1.1*
P-value		0.843	0.008*	0.017*	0.014*	0.008*
Sandy						
	0	93 ± 3	99 ± 6	710 ± 11	6.1 ± 0.2	7.9 ± 0.1
	80	85 ± 5	89 ± 6	748 ± 66	6.3 ± 0.6	8.5 ± 0.1
	1000	96 ± 9	83 ± 3*	943 ± 57*	6.7 ± 0.1*	8.5 ± 0.1
P-value		0.743	0.018*	0.002*	0.020*	0.056
Alum shale						
	0	402 ± 36	170 ± 2	2313 ± 96	17.1 ± 0.8	69.6 ± 0.2
	80	437 ± 30	173 ± 9	2302 ± 41	17.9 ± 0.0	75.6 ± 3.2
	1000	400 ± 25	178 ± 8	2231 ± 27	24.0 ± 1.1*	79.5 ± 0.2*
P-value		0.920	0.039*	0.064	0.007*	0.048*
Submerged						
	0	299 ± 1	267 ± 7	2169 ± 144	26.0 ± 0.9	101 ± 8.4
	80	337 ± 9	261 ± 16	1966 ± 110	27.9 ± 0.8	96 ± 8.4
	1000	308 ± 17	278 ± 23	2584 ± 107*	34.1 ± 0.4*	100 ± 7.1
P-value		0.615	0.242	0.276	0.012	0.831

Table S2. Available micronutrient concentration in investigated soils after 90 days treatment with various supplementations of Si. *P*-value indicates if concentration of element increase/decrease with increased Si-treatment. n=5, ±SE. *indicates significant difference *P*<0.05 from the untreated soil.

Soil type	Treatment Si kg/ha	Cl mg/kg	Mn mg/kg	Fe mg/kg	Zn mg/kg	Cu mg/kg	Mo µg/kg
Clayey							
	0	79 ± 3	18 ± 1.1	2926 ± 73	3.2 ± 0.2	2.0 ± 0.1	128 ± 7
	80	76 ± 5	19.1 ± 1.4	2735 ± 182	3.3 ± 0.1	2.0 ± 0.2	131 ± 2
	1000	80 ± 6	19.6 ± 0.7	3188 ± 206	4.0 ± 0.2*	2.8 ± 0.2*	125 ± 10
P-value		0.855	0.094	0.664	0.031*	0.100	0.689
Sandy							
	0	66 ± 2	14.7 ± 0.7	971 ± 9	1.0 ± 0.0	1.0 ± 0.0	52 ± 0
	80	63 ± 0	14.4 ± 1.3	967 ± 59	1.0 ± 0.0	1.1 ± 0.0	55 ± 3
	1000	65 ± 5	9.9 ± 0.9*	1074 ± 84	1.9 ± 0.1*	1.1 ± 0.1	63 ± 2*
P-value		0.883	0.020*	0.194	0.035*	0.087	0.018*
Alum shale							
	0	182 ± 12	23.1 ± 2.0	4425 ± 57	13.7 ± 0.0	2.0 ± 0.1	95 ± 4
	80	171 ± 6	24.7 ± 0.9	4891 ± 24	12.2 ± 0.1	1.9 ± 0.1	103 ± 4
	1000	191 ± 16	29.8 ± 1.1*	4925 ± 463	15.9 ± 1.0	2.8 ± 0.2*	146 ± 8*
P-value		0.762	0.015*	0.074	0.233	0.008*	0.004*
Submerged							
	0	113 ± 9	26.6 ± 1.8	2727 ± 261	300 ± 4	25.3 ± 1.7	485 ± 13
	80	126 ± 3	26.4 ± 1.8	2585 ± 162	322 ± 7	27.8 ± 0.1	513 ± 37
	1000	129 ± 4	37.4 ± 3.3*	2997 ± 252	310 ± 17	34.5 ± 1.2*	651 ± 49*
P-value		0.115	0.074	0.825	0.795	0.021*	0.009*

Table S3. Biomass of plants grown in nutrient medium during 5-7 days treatment with or without K_2SiO_3. n=5, ±SE. *indicates significant difference *P*<0.05 from the untreated plants.

Plant species	Shoots			Roots			Whole plant			Shoot:whole
K_2SiO_3, (µM)	DW (g)	FW(g)	DW:FW	DW (g)	FW(g)	DW:FW	DW (g)	FW(g)	DW:FW	plant (DW)
Maize										
0	0.700	10.79	0.065	0.577	7.06	0.083	1.275	17.85	0.073	0.548
	(0.252)	(3.97)	(0.002)	(0.077)	(1.48)	(0.013)	(0.273)	(5.21)	(0.007)	(0.089)
5000	0.988	14.27	0.069*	0.644	8.79	0.074	1.632	23.06	0.071	0.605
	(0.251)	(3.34)	(0.002)	(0.041)	(1.08)	(0.006)	(0.274)	(4.32)	(0.002)	(0.056)
Lettuce										
0	5.784	31.27	0.184	1.167	6.33	0.181	2.08	37.60	0.56	0.832
	(1.344)	(9.65)	(0.021)	(0.235)	(1.58)	(0.017)	(0.28)	(11.0)	(0.07)	(0.081)
1000	4.993	26.58	0.187	1.057	5.69	0.185	1.74	32.27	0.59	0.825
	(1.194)	(9.52)	(0.020)	(0.216)	(1.65)	(0.019)	(0.38)	(11.1)	(0.05)	(0.042)
Wheat										
0	2.626	6.69	0.069	0.940	4.33	0.082	0.826	11.02	0.075	0.559
	(0.130)	(1.85)	(0.005)	(0.091)	(0.42)	(0.006)	(0.179)	(2.21)	(0.004)	(0.041)
1000	2.683	6.98	0.075	0.608	4.77	0.090	0.956	11.75	0.081*	0.548
	(0.113)	(0.40)	(0.002)	(0.047)	(0.13)	(0.009)	(0.037)	(0.44)	(0.002)	(0.040)

Table S4. Biomass of plants grown in nutrient medium during three weeks treatment with or without K_2SiO_3. *P*-value indicates if weights increase/decrease with increased Si-treatment. n=5, ±SE. indicates significant difference *P*<0.05 from the untreated plants.

Plant species	Shoot			Root			Whole plant			Shoot: Whole plant
Si, µM	DW, g	FW, g	DW:FW	DW, g	FW, g	DW:FW	DW, g	FW, g	DW:FW	DW
Wheat										
	1.59	17.9	0.089	0.39	5.8	0.068	1.98	23.6	0.084	0.803
0	(0.01)	(0.6)	(0.003)	(0.02)	(0.4)	(0.006)	(0.04)	(0.7)	(0.003)	(0.080)
	1.62	18.0	0.090	0.39	5.7	0.069	2.01	23.7	0.085	0.806
100	(0.13)	(1.8)	(0.011)	(0.01)	(0.7)	(0.008)	(0.07)	(1.5)	(0.006)	(0.073)
	1.67	19.1	0.087	0.39	5.5	0.072	2.06	24.6	0.084	0.811
500	(0.13)	(2.2)	(0.012)	(0.01)	(0.2)	(0.003)	(0.07)	(1.2)	(0.005)	(0.064)
	1.70*	18.8	0.090	0.40	5.7	0.071	2.11*	24.5	0.086	0.806
1000	(0.01)	(2.0)	(0.01)	(0.02)	(0.6)	(0.008)	(0.04)	(1.5)	(0.006)	(0.018)
P-value	<0.001*	0.216	0.846	0.819	0.544	0.387	0.038*	0.607	0.835	0.920
Carrot										
	0.74	7.4	0.100	0.52	5.2	0.100	1.26	12.6	0.100	0.587
0	(0.02)	(0.4)	(0.006)	(0.05)	(0.5)	(0.014)	(0.05)	(0.6)	(0.006)	(0.041)
	0.80	8.1	0.099	0.49	4.8	0.102	1.29	12.9	0.100	0.620
100	(0.08)	(0.5)	(0.011)	(0.05)	(0.4)	(0.012)	(0.07)	(0.5)	(0.007)	(0.032)
	0.77	7.6	0.101	0.53	5.3	0.101	1.30	12.9	0.101	0.592
500	(0.02)	(0.8)	(0.011)	(0.05)	(0.7)	(0.016)	(0.05)	(0.9)	(0.008)	(0.051)
	0.79*	7.8	0.101	0.56	5.5	0.103	1.36	13.3	0.102	0.581
1000	(0.01)	(1.0)	(0.013)	(0.07)	(0.6)	(0.016)	(0.07)	(0.9)	(0.008)	(0.022)
P-value	0.075	0.297	0.557	0.402	0.472	0.737	0.051	0.084	0.341	0.904
Lettuce										
	3.86	20.6	0.188	0.87	4.5	0.193	4.73	25.0	0.189	0.816
0	(0.09)	(2.7)	(0.025)	(0.00)	(0.1)	(0.005)	(0.05)	(1.4)	(0.011)	(0.035)
	3.93	20.4	0.192	0.82	4.3	0.194	4.75	24.7	0.193	0.827
100	(0.34)	(2.4)	(0.028)	(0.04)	(0.0)	(0.010)	(0.19)	(1.2)	(0.012)	(0.058)
	4.05	21.3	0.190	0.83	4.2	0.197	4.88	25.5	0.191	0.830
500	(0.28)	(0.3)	(0.013)	(0.07)	(0.1)	(0.017)	(0.22)	(0.2)	(0.009)	(0.018)
	4.15*	21.7	0.191	0.82	4.3	0.191	4.97*	26.0	0.191	0.835
1000	(0.13)	(2.1)	(0.019)	(0.01)	(0.1)	(0.005)	(0.07)	(1.0)	(0.008)	(0.048)
P-value	0.007*	0.205	0.289	0.044*	0.194	0.862	0.010*	0.182	0.794	0.154
Pea										
	2.78	22.0	0.126	1.28	11.4	0.112	4.05	33.4	0.121	0.686
0	(0.25)	(0.6)	(0.012)	(0.07)	(0.0)	(0.006)	(0.18)	(0.4)	(0.005)	(0.061)
	2.95	23.2	0.127	1.39	12.8	0.108	4.34	36.1	0.120	0.680
100	(0.16)	(0.6)	(0.008)	(0.08)	(1.4)	(0.013)	(0.14)	(1.6)	(0.007)	(0.024)
	3.00	22.7	0.132	1.48	13.9	0.107	4.48	36.6	0.123	0.670
500	(0.28)	(1.9)	(0.017)	(0.09)	(1.9)	(0.016)	(0.21)	(2.4)	(0.010)	(0.025)
	2.91	22.5	0.129	1.47*	13.4	0.110	4.38	35.9	0.122	0.664
1000	(0.25)	(2.5)	(0.018)	(0.10)	(0.2)	(0.008)	(0.19)	(1.6)	(0.008)	(0.045)
P-value	0.176	0.690	0.351	0.032	0.071	0.395	0.220	0.246	0.637	0.666

Table S5. Concentrations of nutrients in shoots and roots of various plant species untreated and treated with K_2SiO_3 during 5-7 days. n = 5 (maize and lettuce) and10 (wheat) ± SE in brackets. * indicates significant difference from the control.

Plant<br>Part<br>Si, µM	N<br>mg g^{-1}	K<br>mg g^{-1}	Mg<br>mg g^{-1}	Ca<br>mg g^{-1}	P<br>mg g^{-1}	S<br>mg g^{-1}	Cl<br>mg g^{-1}	B<br>µg g^{-1}	Mn<br>µg g^{-1}	Fe<br>µg g^{-1}	Zn<br>µg g^{-1}	Cu<br>µg g^{-1}	Mo<br>µg g^{-1}	Si<br>µg g^{-1}
Maize														
Shoot														
0	55.4	19.6	3.1	2.4	1.6	3.4	2.3	3.1	230	264	16.5	9.3	0.18	120
	(1.7)	(0.7)	(0.1)	(0.3)	(0.1)	(0.3)	(0.2)	(0.5)	(13)	(36)	(2.1)	(1.1)	(0.00)	(18)
5000	48.7*	20.1	3.9	2.9	1.1*	3.4	2.1	5.9 *	391*	209	11.8	5.8*	0.15	10294*
	(1.8)	(1.1)	(0.1)	(0.0)	(0.2)	(0.3)	(0.0)	(0.9)	(20)	(6)	(1.1)	(0.1)	(0.01)	(1544)
Root														
0	56.9	15.8	2.7	1.9	2.0	3.8	3.5	4.4	248	198	29.6	10.6	0.09	115
	(1.3)	(1.6)	(0.0)	(0.2)	(0.1)	(0.2)	(0.1)	(0.7)	(1)	(21)	(3.6)	(0.9)	(0.00)	(17)
5000	52.1*	16.0	2.7	1.5	1.6	3.7	3.3	7.5*	381*	173	35.0	18.4*	0.09	14702*
	(1.1)	(1.0)	(0.3)	(0.1)	(0.2)	(0.2)	(0.0)	(1.1)	(48)	(25)	(1.6)	(0.3)	(0.00)	(2205)
Lettuce														
Shoot														
0	43.0	16.8	1.8	3.6	1.7	1.4	2.6	4.9	73	275	121	14.8	0.29	26
	(0.2)	(0.7)	(0.1)	(0.3)	(0.1)	(0.2)	(0.2)	(0.7)	(8)	(32)	(1.5)	(1.9)	(0.04)	(6)
1000	35.7*	16.1	2.0	4.0	1.0*	1.5	2.7	6.8*	94	268	94	12.0	0.30	882*
	(1.5)	(0.9)	(0.1)	(0.5)	(0.0)	(0.1)	(0.3)	(1.0)	(7)	(5)	(0.8)	(0.1)	(0.03)	(132)
Root														
0	46.7	14.9	1.7	2.9	1.9	1.7	4.0	10.6	101	154	158	26.4	0.12	31
	(1.0)	(1.8)	(0.0)	(0.3)	(0.1)	(0.1)	(0.1)	(1.6)	(4)	(22)	(4)	(2.1)	(0.00)	(4)
1000	41.4*	14.3	1.7	2.8	1.6	1.6	3.9	15.6	121*	168	199*	31.8	0.11	1776*
	(1.5)	(1.5)	(0.0)	(0.0)	(0.1)	(0.1)	(0.5)	(2.3)	(1)	(5)	(1)	(1.9)	(0.01)	(266)
Wheat														
Shoot														
0	37.4	12.6	0.9	1.9	2.0	1.6	3.2	2.1	64	157	85	8.2	0.21	24
	(1.1)	(0.8)	(0.04)	(0.2)	(0.02)	(0.1)	(0.2)	(0.2)	(4)	(10)	(0.5)	(0.5)	(0.003)	(1)
1000	31.4*	11.6	1.1	2.0	1.2*	1.8	3.4	3.4*	91*	176	62	6.1	0.23	3535*
	(2.3)	(0.2)	(0.06)	(0.07)	(0.0)	(0.05)	(0.03)	(0.07)	(6)	(8)	(0.1)	(0.6)	(0.02)	(157)
Root														
0	41.3	11.8	0.9	1.5	2.2	1.8	4.8	4.6	92	89	101	14.5	0.09	26
	(2.0)	(0.2)	(0.07)	(0.4)	(0.0)	(0.0)	(0.1)	(0.0)	(4)	(22)	(4)	(2.1)	(0.00)	(5)
1000	39.0	9.6	1.0	1.3	1.6	2.1	4.6	8.3*	117*	111	128*	14.7	0.08	7798*
	(5.0)	(0.1)	(0.0)	(0.0)	(0.0)	(0.0)	(0.5)	(0.0)	(1)	(5)	(1)	(1.9)	(0.01)	(528)

Table S6.

Concentration of various nutrients in shoots and roots of various plant species untreated and treated with silicon for three weeks. *P*-value indicates if concentration of element increase/decrease with increased Si-treatment. n = 4, ± SE in brackets. *indicates significant difference from the control.

Plant Part Si, µM	N $mg\ g^{-1}$	K $mg\ g^{-1}$	Mg $mg\ g^{-1}$	Ca $mg\ g^{-1}$	P $mg\ g^{-1}$	S $mg\ g^{-1}$	Cl $mg\ g^{-1}$	Mn $\mu g\ g^{-1}$	Fe $\mu g\ g^{-1}$	Zn $\mu g\ g^{-1}$	Cu $\mu g\ g^{-1}$	Mo $\mu g\ g^{-1}$	Si $\mu g\ g^{-1}$
Wheat													
Shoot													
	39.4	11.9	1.01	1.9	2.0	1.9	3.4	62.3	159	92.9	8.18	0.21	21
0	(1.1)	(0)	(0.06)	(0.1)	(0.1)	(0.2)	(0.1)	(0.8)	(13)	(7.4)	(0.8)	(0)	(2)
	38.8	11.5	1.05	2	2.1	1.7	3.2	73.4*	176*	68.1*	7.05*	0.216	2486*
100	(0.6)	(1.1)	(0.06)	(0.2)	(0.2)	(0.1)	(0.2)	(5.8)	(4)	(6.2)	(0.08)	(0.013)	(244)
	36.7	11.6	0.96	2	1.8	1.6*	3.5	78*	168	59.1*	5.83*	0.21	3711*
500	(0.7)	(0.2	(0.03)	(0.1)	(0.1)	(0.1)	(0.3)	(5.8)	(14)	(0.3)	(0.15)	(0.007)	(160)
	36.5*	11.4	1.13*	2.1	1.8*	1.6*	3.5	86*	171*	59.2*	6.22*	0.214	4008*
1000	(0.5)	(0.4)	(0.01)	(0.1)	(0)	(0)	(0.2)	(1.4)	(6)	(4.1	(0.3)	(0.007)	(44)
P-value	0.032*	0.331	0.074	0.152	0.048*	0.041*	0.093	<0.001*	0.010*	<0.001*	<0.001*	0.681	<0.001*
Root													
	38.9	11.4	0.94	1.4	2.2	2.7	4.9	102	87	102.7	15.29	0.1	36
0	(5.1)	(0.4)	(0.01)	(0.1)	(0.2)	(0.1)	(0.1)	(5.5)	(8)	(3.9)	(0.1)	(0.005)	(4)
	39.0	9.5*	1.02*	1.4	2.1	2.5	4.7	124.8*	112*	124*	14.47	0.105	3870*
100	(3.1)	(0.4)	(0.02)	(0)	(0)	(0.1)	(0.5)	(3.5)	(4)	(6.1)	(1)	(0.006)	(152)
	38.1	10.1*	0.97	1.4	1.7	2.0*	4.9	117.8*	124*	129.4*	14.09	0.08*	7045*
500	(4.3)	(0.1)	(0.01)	(0.1)	(0.2)	(0.1)	(0.1)	(6.8)	(7	(9.5)	(1.09)	(0.004)	(451)
	37.4	10*	0.94	1.4	1.7	2.0*	4.8	127.4*	115*	136*	14.2*	0.084*	8439*
1000	(2.1)	(0.3)	(0.02)	(0.1)	(0.2)	(0)	(0.4)	(5.5)	(4)	(4.3)	(0.35)	(0.005)	(721)
P-value	0.051	0.040*	0.359	0.894	0.002	<0.001*	0.815	0.009*	0.013*	<0.001*	0.018*	0.025*	<0.001*
Carrot													
Shoot													
	49.8	8.5	0.7	1.5	1.5	1.2	2.4	47.6	113	65.6	5.96	0.146	22
0	(0.2)	(0.6)	(0.02)	(0)	(0.1)	(0.1)	(0.1)	(0.4)	(3)	(6.3)	(0.28)	(0.009)	(2)
	49.9	8.4	0.73	1.4	1.5	1.2	2.4	50	130*	51.1*	4.96*	0.164	158*
100	(4.0)	(0.4)	(0.06)	(0)	(0.1)	(0.1)	(0.1)	(2.7)	(0)	(2.7)	(0.46)	(0.004)	(4)
	47.5	8.6	0.71	1.5	1.4	1.2	2.4	57.5*	127*	46.1*	4.35*	0.16	344*
500	(6.7)	(0.5)	(0.05)	(0.1)	(0.1)	(0.1)	(0.2)	(4.4)	(11)	(0.2)	(0.21)	(0)	(17)
	46.3	8.3	0.81*	1.5	1.3	1.0*	2.4	66.4*	131*	42.3*	4.4*	0.155	586*
1000	(0.3)	(0.4)	(0.07)	(0.1)	(0.1)	(0.0)	(0.2)	(2.7)	(11)	(0.4)	(0.37)	(0.001)	(9)
P-value	0.045*	0.597	0.068	0.744	0.034*	0.100	0.911	<0.001*	0.002*	<0.001*	0.009*	0.117	<0.001*
Root													

0	52.9 (4.6)	8.8 (0.7)	0.68 (0.04)	1.0 (0.1)	1.6 (0)	1.5 (0	3.5 (0.2)	73.5 (2.1)	67 (6)	71.1 (1.1)	10.69 (0.9)	0.072 (0.003)	27 (1)
100	52.8 (7.2)	7.2* (0.4)	0.72 (0)	1.1 (0)	1.5 (0.1)	1.4 (0	3.4 (0.2)	87.6* (4.7)	84 (4)	89.3* (0.8)	10.58 (0.48)	0.074 (0.003)	415* (35)
500	50.5 (7.4)	7.4* (0)	0.69 (0)	1.1 (0.1)	1.3* (0.1)	1.2* (0	3.4 (0.3)	84.7* (2.5)	84 (8)	96* (7)	10.27 (0.41)	0.059* (0.002)	977* (5)
1000	50.3 (5.4)	7.1* (0.3)	0.70 (0.01)	1.3* (0)	1.2* (0.1)	1.2* (0.1	3.5 (0.1)	95.9* (9.2)	85 (6)	95.6* (6.9)	10.48 (0.45)	0.057* (0.002)	1364* (46)
P-value	0.072	0.030*	0.572	0.014*	0.005*	0.009*	0.855	0.011*	0.022*	0.004*	0.124*	<0.001*	<0.001*
Lettuce													
Shoot													
0	46.2 (3.0)	16.7 (0.9)	0.98 (0.04)	2.7 (0.1)	2.7 (0.1)	2.4 (0.2)	4.3 (0.1)	86.3 (5.4)	216 (7)	119.8 (3.4)	10.52 (0.77)	0.277 (0.007)	26 (1)
100	44.9 (0.1)	15.8 (1.3)	1.03 (0.06)	2.7 (0.1)	2.8 (0.2)	2.3 (0.1)	4.4 (0.3)	94.5 (8.2)	230 (15)	97.1* (2.1)	9.28* (0.61)	0.287 (0.012)	96* (7)
500	44.3 (2.2)	16.1 (0.7)	1.01 (0.04)	2.7 (0.2)	2.5 (0.1)	2.3 (0.2)	4.6 (0)	110.2* (3.8)	237* (19)	82.7* (4.1)	8.37* (0.55)	0.301 (0.014)	512* (16)
1000	42.9 (1.0)	15.3* (0.4)	1.15* (0.03)	2.8 (0.2)	2.4* (0.2)	2.1 (0.1)	4.7* (0.3)	121* (11.7)	243* (9)	82.9* (1.5)	8.23* (0.39)	0.305* (0.016)	887* (60)
P-value	0.017*	0.047*	0.048*	0.356	0.038*	0.019*	0.010*	<0.001*	<0.001*	<0.001*	<0.001*	<0.001*	<0.001*
Root													
0	47.3 (1.4)	14.9 (0.6)	1.02 (0.03)	1.7 (0)	2.7 (0.1)	2.7 (0.1)	5.8 (0.4)	116.7 (5.9)	110 (3)	122.3 (11.7)	17.4 (1.53)	0.117 (0.009)	31 (0)
100	47.2 (2.3)	12.2* (0.1)	1.04 (0.04)	1.7 (0)	2.5 (0.2)	2.5 (0)	5.8 (0.1)	144.7* (13.2	137* (4)	149.7* (5.1)	17.88 (1.74)	0.12 (0.007)	762* (9)
500	45.9 (5.7)	11.8* (0.9	1.02 (0.05)	1.8 (0.2)	2 (0.2)	2.2* (0.1)	5.4 (0.1)	138.9* (11.9)	140* (1)	153.1* (12.2)	17.29 (0.6)	0.098* (0.007)	1528* (57)
1000	45.6 (0.5)	12.2* (0.9)	0.98 (0.01)	1.7 (0.1)	2 (0.2)	2.1* (0)	5.5 (0.2)	159.5* (8.9)	147* (13)	160.4* (6.7)	16.23 (0.45)	0.099* (0.004)	1844* (165)
P-value	0.068	0.037*	0.153	0.804	<0.001*	<0.001*	0.046*	0.028*	<0.001*	<0.001*	0.084	0.092	<0.001*
Pea													
Shoot													
0	66.5 (3.6)	17.2 (1.4)	1.47 (0.08)	2.8 (0.1)	2.8 (0.3)	2.3 (0.2)	4.7 (0.3)	93.6 (8)	235 (11)	134.7 (10.7)	11.58 (0.83)	0.294 (0.024)	19 (2)
100	63.3 (7.8)	17.1 (0.3	1.40 (0.07)	2.7 (0)	3.1* (0)	2.5 (0.2)	4.7 (0.2)	104.5 (6.4)	260* (10)	99.8* (1.4)	9.82* (0.41)	0.32 (0.009)	108* (3)
500	62.4 (2.4)	16.5) (1	1.39 (0.06)	2.7 (0.1)	2.8 (0.1	2.5 (0.1)	5.1* (0.2)	114.5* (7.5)	260* (23)	87.5* (2.5)	8.56* (0.21)	0.318 (0.005)	355* (18)
1000	61 (4.8)	17.1 (1.7)	1.63 (0.08)*	2.7 (0)	2.6 (0)	2.6* (0)	5.2* (0.1)	134* (0.7)	255* (2)	87.2* (1.5)	8.54* (0.37)	0.319 (0.014)	641* (16)
P-value	0.036*	0.243	0.216	0.385	0.470	0.033*	0.026*	<0.001*	0.058	<0.001*	<0.001*	0.192	<0.001*

Root													
	62.2	15.6	1.85	1.9	2.4	2.6	5.8	125.8	115	129.1	19.4	0.128	21
0	(0.4)	(0.3)	(0.11)	(0)	(0)	(0)	(0.5)	(6.4)	(1)	(8.1)	(0.37)	(0.000)	(1)
	60.5	12.2*	1.78	1.7*	2.2	2.5	5.8	153.3*	139*	150.8*	19.12	0.123	549*
100	(7.3)	(1)	(0.01)	(0)	(0.2)	(0.2)	(0.5)	(6.5)	(11)	(12)	(1.82)	(0.008)	(48)
	60.7	12.2*	1.87	1.8	2.2	2.0*	6	152.7*	156*	165.1*	17.91*	0.096*	1070*
500	(7.8)	(0.1)	(0.05)	(0.1)	(0.1)	(0.1)	(0.2)	(13.8)	(8)	(13.1)	(0.14)	(0.008)	(71)
	60.2	12.3*	1.80	1.8	2.2	2.0*	6	161.6*	152*	160.7*	17.32*	0.097*	1477*
1000	(1.1)	(0.1)	(0.05)	(0)	(0)	(0.2)	(0.4)	(0.3)	(7)	(14.6)	(1.33)	(0.002)	(48)
P-value	0.078	0.059	0.261	0.125	0.064	0.008*	0.098	0.004*	0.002*	0.031*	<0.001*	0.015*	<0.001*

Table S7

Net accumulation (total uptake of element per g of root DW) of various elements in maize, wheat and lettuce untreated and treated with silicon for 5-7 days. n = 5 (maize and lettuce) or 10 (wheat) ± SE in brackets. * indicates significant difference from the control.

Plant<br>Si, µM	N<br>mg g^{-1}	K<br>mg g^{-1}	Mg<br>mg g^{-1}	Ca<br>mg g^{-1}	P<br>mg g^{-1}	S<br>mg g^{-1}	Cl<br>mg g^{-1}	B<br>µg g^{-1}	Mn<br>µg g^{-1}	Fe<br>µg g^{-1}	Zn<br>µg g^{-1}	Cu<br>µg g^{-1}	Mo<br>µg g^{-1}	Si<br>mg g^{-1}
Maize														
0	12.4	3.96	0.65	0.40	0.49	0.79	0.63	8.1	526	463	49	21	0.30	0.26
	(1.2)	(0.42)	(0.06)	(0.06)	(0.04)	(0.08)	(0.06)	(1.0)	(49)	(64)	(6)	(2)	(0.02)	(0.03)
5000	12.7	4.68*	0.97*	0.60*	0.33*	0.91	0.65	16.5*	980*	517	33 *	27*	0.32	30.49*
	(0.8)	(0.34)	(0.07)	(0.04)	(0.03)	(0.07)	(0.03)	(1.6)	(80)	(40)	(4)	(1)	(0.02)	(3.14)
Lettuce														
0	26.0	9.84	1.09	2.10	1.05	0.91	1.72	34.8	462	1433	757	88	1.55	0.16
	(0.20)	(0.09)	(0.09)	(0.22)	(0.10)	(0.1)	(0.15)	(4.2)	(44)	(175)	(50)	(7)	(0.14)	(0.02)
1000	21.0*	9.03*	1.10	2.20	0.66*	0.88	1.69	47.7*	565*	1517	643*	99	1.52	5.94*
	(0.19)	(0.01)	(0.09)	(0.21)	(0.06)	(0.09)	(0.20)	(5.9)	(49)	(117)	(40)	(10)	(0.16)	(0.74)
Wheat														
0	14.6	4.70	0.36	0.69	0.76	0.64	1.38	10.5	271	528	338	37	0.70	0.10
	(0.9)	(0.61)	(0.33)	(0.10)	(0.04)	(0.01)	(0.15)	(0.98)	(14)	(42)	(20)	(4)	(0.04)	(0.01)
1000	17.7	6.06	0.58	1.01*	0.68*	1.00*	1.98*	23.3	519*	587	303	42	1.10*	23.40*
	(1.1)	(0.42)	(0.01)	(0.06)	(0.02)	(0.09)	(0.21)	(1.31)	(32)	(51)	(28)	(1)	(0.08)	(1.41)

Table S8.

Net accumulation of various elements in wheat, carrot, pea and lettuce untreated and treated with K_2SiO_3 for three weeks. *P*-value indicates if concentration of element increase/decrease with increased Si-treatment. n = 4 ± SE in brackets. Asterisk (*) means significant difference from the control.

Plant Si, µM	N µg/g	K µg/g	Mg µg/g	Ca µg/g	P µg/g	S µg/g	Cl µg/g	Mn µg/g	Fe µg/g	Zn µg/g	Cu µg/g	Mo µg/g	Si µg/g
Wheat													
	199	60	5.1	9.3	10.5	8.1	18.6	355	735	481	48.5	0.95	121
0	(7)	(2.5)	(0.3)	(0.5)	(0.5)	(0.6)	(0.8)	(17)	(54)	(28)	(3)	(0.04)	(9)
	200	57	5.4	9.8	10.8	9	18.1	429	841	406	43.7	1.00	14167*
100	(1)	(3.8)	(0.3)	(0.6)	(0.6)	(0.5)	(1.2)	(25)	(40)	(27)	(2.4)	(0.06)	(951)
	194	59.1	5	9.8	9.3	8.9	19.8	448*	838	380*	38.8*	0.97	22782*
500	(10)	(3.9)	(0.2)	(0.5)	(0.5)	(0.6)	(1.3)	(29)	(57)	(22)	(2.3)	(0.05)	(1328)
	191	57.9	5.7	10.1	9.4	10*	19.6	490*	837	386*	40.4*	0.99	25328*
1000	(11)	(2.6)	(0.3)	(0.5)	(0.4)	(0.4)	(1.3)	(21)	(36)	(20)	(1.8)	(0.05)	(1430)
P-value	0.101	0.752	0.133	0.046*	0.144	0.070	0.201	<0.001*	0.052	0.042*	0.042*	0.086	<0.001*
Carrot													
	124	21.1	1.7	3.2	3.7	3	6.9	142	229	165	19.2	1.01	59
0	(8)	(1.9)	(0.1)	(0.3)	(0.3)	(0.3)	(0.6)	(12)	(19)	(16)	(1.7)	(0.28)	(5)
	134	20.8	1.9	3.3	3.9	3.4	7.2	168	295	172	18.6	1.13	671*
100	(7)	(2)	(0.2)	(0.3)	(0.4)	(0.3)	(0.7)	(17)	(28)	(17)	(2)	(0.34)	(64)
	120	20	1.7	3.2	3.3	3.3	7	168	269	163	16.6	1.31	1478*
500	(6)	(1.8)	(0.2)	(0.3)	(0.3)	(0.3)	(0.6)	(16)	(25)	(14)	(1.5)	(0.29)	(129)
	115	18.8	1.8	3.1	3.1	3.3	6.9	189	269	155	16.7	1,36	2188*
1000	(9)	(2.1)	(0.2)	(0.3)	(0.4)	(0.4)	(0.8)	(20)	(31)	(17)	(2)	(0,28)	(233)
P-value	0.088	0.031*	0.376	0.508	0.069	0.165	0,742	<0.001*	0.106	0.125	0.038	0.684	<0.001*
Lettuce													
	253	89.4	7.1	13.7	14.7	12	24.7	501	1072	656	64.3	1.35	147
0	(15)	(3.3)	(0.3)	(0.4)	(0.4)	(0.6)	(1)	(21)	(27)	(34)	(3.8)	(0.06)	(4)
	261	87.5	7.3	14.6	15.9	13.5	26.6	595	1234	612	62.1	1.49	1219*
100	(9)	(6.6)	(0.5)	(1)	(1.3)	(0.9)	(2)	(53)	(88)	(40)	(5.3)	(0.11)	(87)
	262	90.5	7.8	15.1	14.4	13.5	27.7	677*	1296*	557	58.2	1.57	4030*
500	(13)	(8.4)	(0.7)	(1.6)	(1.4)	(1.2)	(2.3)	(64)	(119)	(53)	(5.2)	(0.14)	(345)
	264	90.2	8.9*	15.8	14.4	14.7*	29.6*	775*	1381*	582	58.1	1.65*	6357*
1000	(18)	(5.7)	(0.5)	(0.8)	(1)	(0.4)	(1.3)	(46)	(72)	(18)	(2)	(0.06)	(380)
P-value	0.130	0.618	<0.001*	0.027*	0.276	<0.001*	<0.001*	<0.001*	<0.001*	0.046	0.082	<0.001*	<0.001*

Pea													
	207	53	4.4	8.1	8.9	7.3	16.1	329	626	422	44.6	0.77	62
0	(16)	(4.1)	(0.3)	(0.6)	(0.7)	(0.6)	(1.4)	(27)	(44)	(35)	(3.4)	(0.06)	(5)
	195	48.4	4.4	8	9.2	7.8	15.7	375	690	362	39.9	0.80	778*
100	(5)	(3.4)	(0.3)	(0.5)	(0.6)	(0.7)	(1.1)	(25)	(49)	(25)	(3)	(0.05)	(56)
	187	45.7	4	7.8	7.9	7.7	16.3	385	683	343	35.3	0.74	1791*
500	(8)	(3.5)	(0.3)	(0.6)	(0.7)	(0.6)	(1.2)	(35)	(60)	(29)	(2.6)	(0.06)	(149)
	181	46.1	4.4	7.5	7.3	7.7	15.6	427	655	333	34.2	0.73	2745*
1000	(11)	(4.1)	(0.3)	(0.6)	(0.5)	(0.6)	(1.3)	(31)	(50)	(29)	(2.9)	(0.06)	(208)
P-value	0.039*	0.060	0.877	0.047*	0.057	0.101	0.681	<0.001*	0.095	<0.001*	<0.001*	0.062	<0.001*
